# Comparative Study of Early- and Mid-Ripening Peach (*Prunus persica* L.) Varieties: Biological Activity, Macro-, and Micro- Nutrient Profile

**DOI:** 10.3390/foods10010164

**Published:** 2021-01-14

**Authors:** Dasha Mihaylova, Aneta Popova, Ivelina Desseva, Nadezhda Petkova, Magdalena Stoyanova, Radka Vrancheva, Anton Slavov, Alexandar Slavchev, Anna Lante

**Affiliations:** 1 Department of Biotechnology, Technological Faculty, University of Food Technologies, 4002 Plovdiv, Bulgaria; 2 Department of Catering and Tourism, Economics Faculty, University of Food Technologies, 4002 Plovdiv, Bulgaria; popova_aneta@yahoo.com; 3 Department of Analytical Chemistry and Physical Chemistry, Technological Faculty, University of Food Technologies, 4002 Plovdiv, Bulgaria; ivelina_hristova_vn@abv.bg (I.D.); magdalena.stoianova@abv.bg (M.S.); radka_vrancheva@yahoo.com (R.V.); 4 Department of Organic Chemistry and Inorganic Chemistry, Technological Faculty, University of Food Technologies, 4002 Plovdiv, Bulgaria; petkovanadejda@abv.bg (N.P.); antons@uni-plovdiv.net (A.S.); 5 Department of Microbiology, Technological Faculty, University of Food Technologies, 4002 Plovdiv, Bulgaria; alslavchev@gmail.com; 6 Department of Agronomy, Food, Natural Resources, Animals, and Environment—DAFNAE, Agripolis, University of Padova, 35020 Legnaro, Italy

**Keywords:** peach, nectarine, biological activity, macro- and micro-nutrient profile, phytochemicals

## Abstract

Exploring the chemical composition and biological activity of different fruit varieties is essential for the valorization of their health claims. The current study focuses on a detailed comparative analysis of three early- and two mid-ripening peach varieties: “Filina” (peach), “July Lady” (peach), “Laskava” (peach), “Gergana” (nectarine), and “Ufo 4” (flat peach). They were characterized in terms of essential nutrients such as carbohydrates (sugars and dietary fibers), amino acid content, and lipids as well as mineral content, fat-soluble vitamins, carotenoids, and chlorophyll. Polyphenolic compounds and the related antioxidant activity were also assessed. The methanolic extract of the peel seems to be richer in the studied biologically active substances compared to the fleshy part of the fruit. Anthocyanins were most abundant in “Gergana” and “July Lady” extracts (6624.8 ± 404.9 and 7133.6 ± 388.8 µg cyanidin-3-glucoside/100 g fw, resp.). The total phenol content of the samples varied from 34.11 ± 0.54 to 157.97 ± 0.67 mg gallic acid equivalents (GAE)/100 g fw. “Filina” and “July Lady” varieties possessed the highest antioxidant activity. Overall, the results of this study confirm that the studied peach varieties have satisfactory nutritional value and are potential sources of biologically active substances. Each variety represents an individual palette of nutrients that should be considered separately from the other.

## 1. Introduction

Nutrition can be seen as balanced when eating the appropriate amounts of a large variety of foods [1]. Fruits are an important part of the human diet. It has been widely reported that a menu rich in fruits and vegetables can reduce the risk of chronic disease such as cancer and cardiovascular disease [2,3]. The World Health Organization (WHO) promotes an intake of fruits and vegetables of about 400 g/day. The recommended dietary allowance (RDA) of different nutrients shows the daily average dietary intake that meets the needs of nearly all healthy members of a particular life stage and gender group [4].

Stone fruits are highly prized for their unique aesthetic and organoleptic characteristics. Traditionally, fruit quality indicators include appearance, sugar, and acid content. However, fruits also contain innumerable phytochemicals, which, although at relatively low concentrations, play a key role in overall quality [5,6]. Some of these substances can be major factors for their color and aroma [7]. In addition, many of these compounds have been found to play a protective role against certain diseases [8]. When taken regularly and in significant amounts as part of the daily diet, these metabolites can have noticeable long-term physiological effects [9].

*Prunus persica* L. Batsch (peach) is the second most important fruit in Europe after the apple [10]. Italy and Spain are the top European Union (EU) producers. The fruit of *Prunus persica* is suitable for direct consumption, possessing a pleasant and refreshing taste. Peaches are traditionally cultivated crops in Bulgaria. Due to the support of different EU funds, the amount of stone fruit gardens continues to grow. Stone fruits are Bulgaria’s largest fruit category, accounting for 40 percent of Bulgarian total fruit production. Since 2017, Bulgaria has ranked fifth in the EU in the area of peach orchards according to Eurostat data. There are plenty of Bulgarian varieties with excellent properties that could be presented to the EU market [11].

The nutritional profile of peaches depends on the presence of organic acids, minerals, carbohydrates, and dietary fiber that are among the major constituents of the fruit [12]. Apart from their sensory quality, peaches are considered a good source of valuable substances that can promote positive health effects when consumed. One of these substances is phenolic compounds that comprise phenolic acids, flavonoids, and anthocyanins.

Information about the nutritional value of peach fruit from Bulgaria in different ripening stages and varieties is scarce. Moreover, three of the studied varieties have been relatively recently introduced. In this context, the present paper aims at characterizing and comparing three early- and two mid-ripening peach varieties: “Filina” (peach), “Ufo 4” (flat peach), “Gergana” (nectarine), “July Lady” (peach), and “Laskava” (peach) first, and then making propositions about the most beneficial variety in terms of nutrient quality. They were specified in terms of essential nutrients, such as carbohydrates (sugars, and dietary fibers), proteins (including amino acid content), and lipids as well as fat-soluble vitamins, carotenoids, and chlorophyll. Mineral content was also determined. In addition, polyphenolic compounds and the related antioxidant activity were studiedby Ferric Reducing Antioxidant Power (FRAP), Cupric Reducing Antioxidant Capacity (CUPRAC), 2,2-Diphenyl-1-picrylhydrazyl (DPPH) and 2,2′-Azinobis-(3-Ethylbenzothiazoline-6-Sulfonic Acid (ABTS) methods.

## 2. Materials and Methods

### 2.1. Plant Material

**Figure 1:**
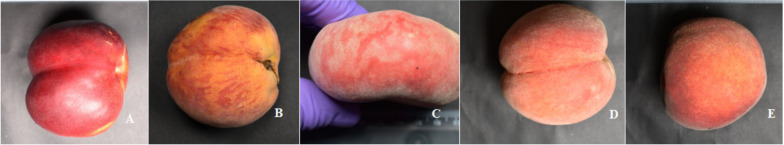
Early- and mid-ripening peach (*Prunus persica* L.) varieties: (**A**) “Gergana”, (**B**) “Filina”, (**C**) “Ufo 4”, (**D**) “July Lady”, (**E**) “Laskava”.

The following early-ripening peach and nectarine varieties were used: “Filina” (peach), “Ufo 4” (flat peach, white flesh), and “Gergana” (nectarine). “Filina” (Figure 1B) is a Bulgarian variety, a result of the breeding selection of “Maycrest” × “July Lady”. “Gergana” (Figure 1A) is also a variety created in Bulgaria by combining Goldengrand and Aureliogrand varieties. “Ufo 4” (Figure 1C) is an Italian variety, part of the Ufo series, containing 9 varieties, from 1 to 9, created by the crossing of “Maybelle” × “Saturn”.

The mid-ripening “Laskava” (peach) and “July Lady” (peach) were investigated in the present study. “Laskava” (Figure 1D) is a Bulgarian variety, created by cross-species hybridization, with the participation of the species *Prunus persica* (L.) Batsch and *Prunus ferganensis* (Kost. and Rjab.) from the parent combination “Hale” × (“Elberta” × “Fergana Yellow”). “July Lady” is an American peach variety spread worldwide (Figure 1E).

The trees were grafted on seedlings and were planted in the springs of 2012–2013 at two densities—50 and 100 trees per ha. The varieties were clingstone and semi-clingstone types. No bactericides were applied to plantings during testing.

The undamaged peach, nectarine, and flat fruit were harvested at eating ripeness in the Fruit-growing Research Institute, Plovdiv, BG (lat. 42.10384828045957 and long. 24.72164848814686). Fruit were considered ripe, on the trees, when the growth of the fruit had stopped, the fruit began softening, exhibited a yellow or orange ground color (which is also representative for each variety), and was easily detached. Extraction procedures were performed and described for each analysis. The moisture content of the peach samples in fresh state was as follows: “Filina” 84.57%, “Gergana” 84.74%, “Ufo 4” 87.03%, “July Lady” 84.57%, and “Laskava” 85.26%.

### 2.2. Determination of Mineral Composition

Peach fruit (peel and pulp) was analyzed in a fresh state for macro- and microelements and heavy metals by the microwave mineralization method. The analysis was performed in the accredited laboratory complex of the Agricultural University, Plovdiv. Trace elements have been determined by atomic absorption spectrometry after ash drying according to EN 14082:2003 [13]. Sodium, potassium, calcium, and magnesium contents were determined by atomic absorption spectrometry following EVS-EN 1134:2000 [14].

### 2.3. Amino Acid Analysis

The amino acid composition was determined by the method described by Tumbarski et al. [15]. Fresh peach samples were subjected to acid hydrolysis using 6N HCl for 24 h at 105 °C. An aliquot of the hydrolysate was derivatized using an AccQ-Fluor reagent Kit (Waters). The derivate was separated on an RP AccQ-Tag™ silica-bonded amino acid column C18, 3.9 mm × 150 mm (Waters) conditioned at 37 °C using an ELITE LaChrom HPLC system (VWR™ Hitachi, Tokyo, Japan). A sample of 20 μL was injected and the elution of the amino acids was performed by a gradient system: eluent A, buffer WAT052890 (Waters) and eluent B, 60% acetonitrile (Sigma-Aldrich, Merck, Darmstadt, Germany) with a constant flow rate of 1.0 mL/min [15]. The amino acids were detected using a diode array detector (DAD) at 254 nm. The amino acid peaks were then analyzed using EZChrom Elite™ software and were calculated based on the amino acid standard calibration curve (amino acid standard H, Thermo Fisher Scientific, Waltham, MA USA). The results are expressed as mg AA/100 g fresh weight (fw).

### 2.4. Chemical Analysis

Total nitrogen content was determined using the Kjeldahl method according to ISO 1871 and protein content was calculated by multiplying the result by a conversion factor of 6.25.

### 2.5. Carbohydrate, Lipid, and Fiber Analysis

The preparation of sample extracts was performed with distilled water (solid to liquid ratio 1:5 (*w*/*v*)) in an ultrasonic bath (VWR, Malaysia, Singapore) with a frequency of 45 kHz and 30 W power at 45 °C in triplicate. The samples were filtered. The contents of sugars and sorbitol were determined using a Shimadzu HPLC, coupled with an LC-20 AD pump, and a Shimadzu RID-10A refractive index detector (RID). The separation was done on a Shodex^®^ Sugar SP0810 (300 mm × 8.0 mm i.d.) column with Pb^2+^ and a Shodex SP-G guard column (5 μm, 6 mm × 50 mm) (Shodex Co., Tokyo, Japan) operating at 85 °C. The mobile phase was ultra-purified water (Water purification system Adrona B30 Integrity+HPLC, Riga, Latvia) with a flow rate of 0.5 mL/min. The injection volume was 20 μL [16].

The total carbohydrate content of the samples was calculated by:

Total carbohydrates, % = 100 − (moisture, %) − (ash, %) − (protein, %) − (lipids, %).(1)

Lipid content was determined according to the Association of official analytical collaboration (AOAC) methods (2012) using Soxhlet apparatus. Each sample (around 2 to 3 g) was packed in a pre-weighed, oven-dried thimble. The thimbles were stapled and placed in a Soxhlet apparatus, and extracted for 6 h with n-hexane. The extracts were evaporated under vacuum and the residues were weighed. The results are expressed as g/100 g fw.

The total dietary fibers were determined using a K-TDFR-100A (Megazyme, Ireland), according to AOAC method 991.43 [17] “Total, soluble and insoluble dietary fibers in foods” (First action 1991) and American association of cereal chemistry (AACC) method 32-07.01 “Determination of soluble, insoluble and total dietary fibers in foods and food products” (final approval 10-16-91). Total chlorophylls were spectrophotometrically determined in 95% ethanol extracts at three wavelengths (664, 648, and 470 nm) and calculated according to Lichtenthaler and Wellburn [18]. The results are presented as g/kg fw. 

### 2.6. Extraction of Phenolic Compounds

Three extraction procedures with respect to phenolic compounds were carried out as follows:

Homogenized fresh whole fruit (peel and pulp) from each variety (each 20 g) cut into small pieces was extracted with 80% aqueous methanol (methanol:water, 80:20, *v*/*v*) at 50 °C by ultrasonication for 30 min (MEF). The residues and the extracts were separated by filtering through a filter paper; the obtained residues were re-extracted with a fresh portion of extractant in the same conditions.

The peel of fresh fruit cut into small pieces (each 15 g) was extracted with 80% aqueous methanol (methanol:water, 80:20, *v*/*v*) at 50 °C by ultrasonication for 30 min (MEP). The residues and the extracts were separated by filtering through a filter paper; the obtained residues were re-extracted with a fresh portion of extraction solvent in the same conditions.

Homogenized fresh whole fruit (peel and pulp) from each variety (each 20 g) cut into small pieces was extracted with 100 mL water by ultrasonication at 50 °C for 15 min (WEF). The extract was then subjected to heat reflux extraction for 30 min and afterwards the residues and the extracts were separated by filtering through a filter paper.

The extracts recovered from each of the extraction procedure were subjected to removing the excess of the solvent by distilling off in a vacuum rotary evaporator (IKA RV10 digital, IKA HB 10 digital water bath -IKA^®^-Werke GmbH & Co., Germany) at 50 °C. The obtained semi-liquid extracts were preserved at 4 °C, until used for further experiments.

### 2.7. Identification and Quantification of Phenolic Acids

The qualitative and quantitative determination of phenolic acids in the extracts was performed by using a Hitachi LaChrom Elite^®^ HPLC System (Hitachi High Technologies America, Inc., Schaumburg, Illinois, USA), coupled with diode-array detector (DAD, L-2455) and EZChrom Elite™ software . Separation of the phenolic acids was performed by a Supelco Discovery HS C18 column (5 μm, 25 cm × 4.6 mm), operated at 30 °C under gradient conditions with mobile phase consisting of 2% (*v*/*v*) acetic acid (solvent A) and acetonitrile (solvent B), as reported by Mihaylova et al. [19]. The gradient program used was: 0–1 min: 95% A and 5% B; 1–40 min: 50% A and 50% B; 40–45 min: 100% B; 46–50 min: 95% A and 5% B. The detection of phenolic acids was carried out at 280 nm for gallic, protocatechuic, and cinnamic acids and at 320 nm for chlorogenic, caffeic, ferulic, p-coumaric, sinapic, rosmarinic, and chicoric acids at a flow rate of 0.8 mL min. The results are expressed in µg/g fw.

### 2.8. Carotenoid Content

Freeze-dried plant material (0.2 g) was extracted with 4 mL methanol (1:20, *w*/*v*), and then a 5 mL tetrachlormethane:methanol mixture (3:1, *v*/*v*) with 0.5% butylated hydroxytoluene added. After the extraction in an ultrasonic bath (VWR, USC200T, 60 W, 45 kHz, Magna Park Lutterworth, Leicestershire LE17 4XN, England, 60 W, 45 kHz) for 15 min, 1 mL of 10% NaCl was added. The samples were centrifuged, and the tetrachlormethane fraction was separated, filtered through anhydrous Na_2_SO_4_, and used for carotenoid analysis. Qualitative and quantitative determination of carotenoids was performed by using a LaChrom Elite (Hitachi, Tokyo, Japan) high-performance liquid chromatography (HPLC) system equipped with a diode array detector (DAD) and LaChrom Elite (Hitachi, Tokyo, Japan) software. The assay was performed according to the method described by Mihaylova et al. [20] with some modifications. Separation of the carotenoids was performed on a Supelco Discovery HS C18 column (Sigma-Aldrich, Darmstadt, Germany, 5 μm, 25 cm × 4.6 mm), at 30 °C with a 1 mL/min flow rate of mobile phase consisting of methanol:acetonitrile (8:2, *v*/*v*, solvent A) and tert-butyl methyl ether (MTBE, solvent B). The gradient elution program was performed as follows: 0–0.5 min 95% solvent A, at 3 min 80% solvent A, from 4.5 to 10 min 65% A/35% B, and at 20 min 95% A/5% B. The detection of β-carotene was carried out at 450 nm and the detection of lutein and lycopene at 470 nm. The results are expressed as μg/g fw, according to the established percentage of moisture content for each peach variety.

### 2.9. Tocopherol Content

Freeze-dried plant material (1 g) was saponified with a 10 mL solution (0.1 g NaCl, 4.0 g KOH, and 0.5 mg of BHT dissolved in 96% ethanol in a 50 mL volumetric flask) in a water bath at 70 °C under reflux for 30 min. After the saponification process, 15 mL 1% NaCl and a 15 mL mixture of n-hexane and ethyl acetate (9:1, *v*/*v*) were added. The organic phase was separated, vacuum evaporated to dryness, and then dissolved in 1 mL HPLC grade methanol (Sigma) for further analyses.

Separation of tocopherols was performed on a Symmetry^®^ C18 (5 μm, 15 cm × 4.6 mm) column (Waters, Milford, CT, USA) at 30 °C in isocratic mode with a mobile phase of methanol:water (98:2, *v*/*v*) with a flow rate of 2 mL/min [20]. The tocopherols were detected with a DAD at 285 nm. The results are expressed as μg/g fw, according to the established percentage of moisture content for each peach variety.

### 2.10. Determination of Total Polyphenolic Content (TPC)

The TPC was analyzed following the method of Kujala et al. [21] with some modifications. Each extract (0.1 mL) was mixed with 0.5 mL Folin–Ciocalteu reagent and 0.4 mL 7.5% Na_2_CO_3_. The mixture was vortexed and left for 5 min at 50 °C. After incubation, the absorbance was measured at 765 nm. The TPC is expressed as mg gallic acid equivalents (GAEs) per 100 g fw.

### 2.11. Determination of Total Flavonoid Content (TFC)

The total flavonoid content was evaluated according to the method described by Kivrak et al. [22]. An aliquot of 0.5 mL of the sample was added to 0.1 mL of 10% Al(NO_3_)_3_, 0.1 mL of 1 M CH_3_COOK, and 3.8 mL of ethanol. After incubation at room temperature for 40 min, the absorbance was measured at 415 nm. Quercetin (QE) was used as a standard and the results are expressed as mg quercetin equivalents (QE)/100 g fw.

### 2.12. Determination of Total Monomeric Anthocyanin Content

The total monomeric anthocyanin content was determined using the pH differential method [23]. Properly diluted samples were mixed with KCl (0.025 M, pH 1.0) and CH_3_COONa (0.4 M, pH 4.5) with an appropriate dilution factor. Absorbance (A) was measured using a UV–Vis spectrophotometer at 520 and 700 nm after a 15 min incubation at room temperature, and the results were calculated as follows:

A = (A_520_ − A_700_)pH 1.0 − (A_520_ – A_700_)pH 4.5 (2)

The monomeric anthocyanin (MA) pigment concentration in the samples was calculated as:

Monomeric anthocyanin pigment (mg/liter) = (A × MW × DF × 1000)/(ε × 1)(3)

where M represents the molar mass of cyanidin-3-glycoside (449.2 g/M), DF is the dilution factor, ε is molar extinction coefficient (26,900 L/M × cm), and 1 is the cuvette optical path length (10 mm). The final anthocyanin concentration is expressed as µg cyanidin-3-glucoside (C3GE)/100 g fw.

### 2.13. Determination of Antioxidant Activity

#### 2.13.1. DPPH^•^ Radical Scavenging Assay

The ability of the extracts to donate an electron and scavenge 2,2-diphenil-1-picrylhydrazyl (DPPH) radicals was determined by the slightly modified method of Brand-Williams et al. [24] as described by Mihaylova et al. [25]. A freshly prepared 4 × 10^−4^ M solution of DPPH was mixed with the samples in a ratio of 2:0.5 (*v*/*v*). The light absorption was measured at 517 nm after a 30 min incubation. The DPPH radical scavenging activity is presented as a function of the concentration of Trolox—Trolox equivalent antioxidant capacity (TEAC) and is defined as the concentration of Trolox with equivalent antioxidant activity expressed as μM TE/100 g fw.

#### 2.13.2. ABTS^•+^ Radical Scavenging Assay

The radical scavenging activity of the extracts against 2,2´-azino-bis(3-ethylbenzothiazoline-6-sulfonic acid) (ABTS^•+^) was estimated according to Re et al. [26]. Briefly, ABTS radical cation (ABTS^•+^) was produced by reacting ABTS stock solution (7 mM) with 2.45 mM potassium persulfate (final concentration) and allowing the mixture to stand in the dark at room temperature for 12–16 h before use. Afterward, the ABTS^•+^ solution was diluted with ethanol to an absorbance of 0.7 ± 0.02 at 734 nm and equilibrated at 30 °C. After the addition of 1.0 mL of diluted ABTS^•+^ solution to 0.01 mL of samples, the absorbance reading was taken at 30 °C after 6 min. The results are expressed as the TEAC value (μM TE/100 g fw).

#### 2.13.3. Ferric-Reducing Antioxidant Power (FRAP) Assay

The FRAP assay was carried out according to the procedure of Benzie and Strain [27] with slight modification. The FRAP reagent was prepared fresh daily and was warmed to 37 °C prior to use. One hundred and fifty microliters of plant extracts were allowed to react with 2850 µL of the FRAP reagent for 4 min at 37 °C, and the absorbance was recorded at 593 nm. The absorbance was recorded at 593 nm and the results are expressed as μM TE/100 g fw.

#### 2.13.4. Cupric Ion-Reducing Antioxidant Capacity (CUPRAC) Assay

The CUPRAC assay was carried out according to the procedure of Apak et al. [28]. One milliliter of CuCl_2_ solution (1.0 × 10^−2^ M) was mixed with 1 mL of neocuproine methanolic solution (7.5 × 10^−3^ M), 1 mL of CH_3_COONH_4_ buffer solution (pH 7.0), and 0.1 mL of herbal extract (sample) followed by the addition of 1 mL of water (total volume = 4.1 mL) and mixed well. Absorbance against a reagent blank was measured at 450 nm after 30 min. Trolox was used as a standard and the results are expressed as μM TE/100 g fw.

### 2.14. Protein Quality Calculations

The amino acid score (AAS) was determined by comparing the amino acid (AA) composition of each test article with the recommended FAO/WHO reference pattern, mg/g protein [29]. Both the composition and reference patterns were first expressed in mg AA/g protein units. The lowest calculated AA ratio (limiting AA) was considered as the AAS [30].

### 2.15. Statistical Analysis

Analytical determinations were performed in triplicate and the results are expressed as mean ± SD. Relevant statistical analyses of the data were performed by one-way ANOVA and a Tukey–Kramer post hoc test (α = 0.05), as described by Assaad et al. [31]. Principal component analysis (PCA) was applied after unit variance scaling to the data (SIMCA-P version 14.1; Umetrics, Umeå, Sweden).

## 3. Results and Discussion

Minerals are an important part of plant metabolism. Based on their role, they can be divided into macro- and microelements. In this regard, fruits are an essential source of minerals. In the present study, the total mineral content was evaluated. It varied from 689.74 to 1271.17 mg/kg fw (Table 1.). Early-ripening varieties “Filina” and “Gergana” seem to be the richest in mineral elements (1271.17 mg/kg fw and 1121.03 mg/kg fw, resp.). At the same time, the early-ripening flat peach “Ufo 4” and the mid-ripening peach variety “Laskava” had the lowest values of mineral elements (689.74 and 899.7 mg/kg fw, resp.). Cu, Fe, Mn, Cr, and Zn are the microelements found in the studied peach varieties. These elements are the most abundant in “Filina” followed by the “Laskava” variety. Copper absorption primarily occurs in the small intestine via both saturable mediated and nonsaturable nonmediated mechanisms and its RDA is 1700 μg/day, which shows that the currently studied peach varieties can be a moderately sufficient source of copper in the daily diet. The recommended daily intake of iron is 0.35 mg/kg. All of the studied peach varieties could account for a small part of the Fe daily intake. Chromium concentration in all the peach varieties was below the maximum permissible limit value (2.3 mg/kg) [32]. Among the macroelements, potassium is definitely predominant in all studied varieties. Its intake is positively associated with bone metabolism, lower blood pressure, and reduced cardiovascular disease morbidity and mortality [33,34]. “Filina” and “July Lady” possessed the highest content (1036.78 and 1179.00 mg/kg fw, resp.), while “Ufo 4” and “Laskava” had the lowest (574.67 and 701.29 mg/kg fw). Potassium is a major intracellular cation in the body, and its RDA is between 0.4 and 5.1 g/day. All of the studied peach varieties can be accountable for a considerable part of the K daily intake, as peaches have been previously reported as a good source of this microelement [35]. Together with calcium and magnesium, potassium participates in amino acid and therefore protein synthesis [36]. The RDA for sodium, in particular, varies from 1.0 to 2.3 g/day. Although most of this quantity is added in different foods by cooking, the studied peach varieties can contribute to the daily RDA. The daily magnesium intake varies from 3.5 to 6.0 mg/kg according to age group, which means that only “Filina” and “Laskava” can relatively contribute to the daily Mg intake. Phosphorus is the second most abundant mineral in the studied peach varieties, with the exception of “July Lady”, where it was absent. This is in agreement with the USDA [37] and BEDCA [38] databases. The adult requirements for phosphorus are based on studies of serum inorganic phosphate concentration, and the content of the average adult diet for both men and women is about 380 to 1055 mg/day [39], which gives reason to conclude that the studied peach varieties cannot be seen as P sources. In all varieties, lead was under 0.1 mg/kg fw. Nitrogen content was also studied by the method of Kjieldhal. It varied between 1.3 and 9.6 g/kg fw. In these values, minor amounts of protein nitrogen are also included.

Amino acids are the building elements of proteins. They may also occur in free form or in non-protein compounds [40]. Apart from their nutritional value in fruits, amino acids also contribute to their taste [41]. The results of the amino acid composition of the studied fruit varieties are presented in Table 2. Nine essential amino acids were determined in all samples. “July Lady” fruit was the poorest in total essential amino acids—15.17 mg/100 g fw, while the “Ufo 4” fruit was the richest—733.93 mg/100 g fw. The same trend was maintained for the total content of amino acids. The total amino acid content varied from 31.51 to 1277.59 mg/100 g fw in the studied varieties.

It is well known that most fruits do not have enough protein and the quality in terms of essential amino acid content is low [42]. The amino acid score method was used to evaluate the protein quality of the studied peach varieties following the method described by Millward [43]. As evident from Table 2, the studied peach varieties cannot be considered to have high protein density. The amounts of essential amino acids are quite limited and they cannot contribute to providing the necessary amounts of quality protein for adults (Table 3). All of the amino acids are limiting. “Ufo 4” and “Gergana” have better amino acid scores compared to “Filina”, “Laskava”, and “July Lady”. The results are comparable to the ones documented by Botoran et al. [44] concerning the amino acid profile of selected fruits, including peaches from unknown varieties. In addition, Farina et al. [45] found that varieties with large and attractive fruit often lacked real nutritional quality.

Table 4 visually presents the results with respect to carbohydrate, dietary fiber, and total lipid content. Total carbohydrates in the investigated peach fruit varied from 6.54 g/100 g fw to 12.42/100 g fw. Glucose, fructose, sucrose, and polyol sorbitol were the sugars detected in the samples. The presence of the abovementioned sugars was previously reported by Farina et al. and Colarič et al. [46,47] for different peach and nectarines cultivars. Sucrose was the predominant sugar among the investigated carbohydrates, as its values were highest in “Laskava”—4.71 g/100 g fw, followed by “July Lady”—2.63 g/100 g fw. Therefore, these two varieties should be considered the sweetest. The “Laskava” variety contained the highest values of the monosaccharides glucose (1.51 g/100 g fw) and fructose (0.86 g/100 g fw), as well as polyol sorbitol—0.11 g/100 g fw. In this case, contrary to the statement of Nowicka et al. [47], the glucose content was higher than that of fructose in every analyzed variety. The glucose/fructose ratio was 1.7–1.8 for three peach varieties (“Filina”, “Gergana”, and “Laskava”), while in the other two (“Ufo 4” and “July Lady”), it was above 2.0 (Table 4). The sucrose/glucose ratio was above 2.3 and reached 4.66, which points out that the sweetness of peaches is mainly due to sucrose, and not so much to glucose and fructose.

The sorbitol content in the investigated peach varieties was the lowest among all carbohydrates. Its concentration ranged from 0.2 to 0.11 g/100 g fw. In three of the peach varieties (“Filina”, “Gergana”, and “Ufo 4”), the sorbitol concentration was the lowest—0.2 g/100 g fw. Sorbitol, together with sucrose, is the main transport sugar —a product of photosynthesis in the leaves—which is not produced in peach fruit, but is transferred from other parts of the tree by the phloem [48]. “Filina” and “Gergana” varieties contained close glucose, fructose, and sorbitol values. It is considered that peach fruit with higher fructose contents are firmer and have good flavor [46]. Therefore, based on this statement, the “Laskava” variety could be considered as the most flavorful, delicious, and aromatic peach among the investigated samples.

Total sugar content was the highest in the “Laskava” variety (7.19/100 g fw) and the lowest in the “Gergana” variety (1.85 g/100 g fw). The total sugar values correspond well to the ones reported for other peach varieties (4.6–9.6%) [46]. In terms of total sugars, the “Laskava” variety is comparable to some cultivars, i.e., “Romestar”, “Anita”, “Orion”, “Venus”, “Maria Laura”, and “Weinberger” [46]. Moreover, the sugar content in the “Laskava” variety was compatible with the glucose, fructose, and sucrose values reported for the “Zaolupantao” flat peach [49].

Overall, the total sugars did not exceed 72 g per kg fruits in the investigated peach varieties, which corresponds to the statement for early and late cultivars reported by Colarič et al. [46]—a maximum of 100–110 g of total sugars per kilogram, contributing to the desirable flavor.

The energy value of each variety was calculated as well and the results show that the highest in energy was the mid-ripening variety “Laskava” (280 kJ), while “Ufo 4” (165 kJ) had the lowest energy value. “Laskava” was the variety with the highest lipid content, which considerably contributes to its energy capacity. All fruit varieties can account for a small amount of the daily energy intake, namely, between 2 and 3%. Most of the energy is at the expense of the carbohydrates contained in the studied fruit. Carbohydrates are the main energy sources for living organisms; thus, the currently investigated peaches can provide a small portion of easily accessible daily energy as well as some other important nutrients.

Dietary fiber represents carbohydrates that are not digested or absorbed in the small intestine. They are thought to play an important role in preventing diseases such as cardiovascular disease, colorectal cancer, lung cancer, obesity, and type 2 diabetes [50]. Their content decreases during ripening due to cell wall hydrolysis by cellulolytic enzymes [51]. In this study, the total, insoluble, and soluble dietary fiber contents of peach samples were established. They ranged as follows: 2.05 ± 0.08–3.18 ± 0.10 and 1.49 ± 0.07–2.35 ± 0.07 and 0.52 ± 0.04–0.82 ± 0.04 g/100 g fw, respectively. The current results are in a close agreement with the ones of Leontowicz et al. [52]. Although the amount of fiber in 100 g of peaches was lower than the suggested dietary target of 25 g per day defined by international guidelines [53], the studied peach varieties are still an excellent source of dietary fiber. According to Regulation (EC) No. 1924/2006 [54] food systems containing at least 3 g of fiber per 100 g or at least 1.5 g of fiber per 100 kcal could be claimed to be a source of fiber. Thus, the “Gergana” variety, distinguished by having a dietary fiber content above 3 g/100 g fw, can be considered as a source of fiber. The soluble fiber fraction is thought to possess various beneficial effects, such as gastrointestinal protection, blood pressure reduction, and cholesterol level reduction [55]. Dietary fiber remains in the residues of juice processing and is associated with good water- and oil-holding capacity [56]. In this regard, peach juice by-products were evaluated as a valuable source possessing up to 13% soluble dietary fiber [57]. They come mainly from the peach peel. In the currently established results, “July Lady” had the highest content of SDF—0.82 ± 0.04 g/100 g fw.

The lipid content in the analyzed peaches ranged from 0.67 g/100 fw to 2.58 g/100 g fw. The highest lipid content was detected in the “Laskava” variety, which was 3.8 times the lipid content of “Gergana”. The crude lipid contents of many edible fruits are usually lower than 2%, and vary considerably depending on the climate, variety, geographical origin, harvest year, and the methods of cultivation [58]. Lipids are necessary for the absorption of fat-soluble vitamins and act as cofactors for the proper digestion of certain nutrients.

Total carotenoid content varied between 2.53 and 42.24 µg/g fw and the highest values were established in the “Gergana” variety (Table 5). Among the studied carotenoids, lycopene was evaluated to possess the highest content in the “July Lady” variety—23.85 µg/g fw. At the same time, the flat peach variety (“Ufo 4”) had 0.89 µg/g. β-carotene was identified with a high concentration only in “Gergana” (6.50 µg/g fw) and “July Lady” samples (14.54 µg/g fw). δ-tocopherol and γ-tocopherol were not detected in any of the samples. Equally, Dabbou et al. [35] reported a predominant presence of β-carotene in various peach varieties and Gil et al. [59] disclosed that β-carotene (provitamin A) and small quantities of α-carotene and β-cryptoxanthin are present in some peach cultivars. However, peaches and nectarines are considered to possess moderate levels of carotenoids and phenolics, but due to their high consumption either fresh or processed, they ultimately contribute significantly with dietary bioactive compounds [60].

The tocopherol content was attributed to the presence only of α-tocopherol and there was a lack of δ-tocopherol and γ-tocopherol (Table 5). The content of α-tocopherol, which is the most active form of vitamin E, was established to be in the 2.32 to 60.40 µg/g fw range. According to Ariel et al. [60], α-tocopherol content in Prunus fruits varied from 0.07 to 26 mg/100 g, which corresponds to the current results.

The presence of chlorophyll is an indicator of fruit maturity. Its content decreases during the ripening of the fruit and disappears completely at full maturity at the expense of other colored compounds like carotenoids, anthocyanins, etc. [41]. Among the studied samples, the flat peach variety “Ufo 4” was the only one possessing chlorophyll. A total of 76.28 mg/g fw was measured.

Antioxidant compounds have always been an attractive research area. Phytochemicals are among the most extensively studied. In peaches and nectarines, they represent mainly phenolics and carotenoids. Polyphenols are plant secondary metabolites that have a protective role against environmental stress [61]. Their concentration varies in different parts of the fruit, moreover, wide inter-varietal differences exist [62]. Nevertheless, fruit and vegetables are the major sources of phenolic compounds in the human diet [63]. Moreover, fruit phenolics have a role in fruit visual appearance (color), taste (astringency), and health-beneficial antioxidant properties [62]. These biologically active substances are believed to have several health benefits, as it is recommended to consume more than 600 mg/day polyphenols in a healthy diet rich in fruits and vegetables [64]. A recent study concluded that the middle, lower, and upper quartiles of polyphenol intake were 326, 167, and 564 mg/day in European adolescents [65]. In the present study, total phenolic, flavonoid, and anthocyanin contents, as well as the individual phenolic acids, were evaluated and the results are presented in Table 6. Three different extracts were studied, aiming at interpreting a detailed phytochemical profile of *P. persica* varieties. Total phenolic content in the methanolic peel extracts varied between 157.97 ± 0.67 for “July Lady” and 78.19 ± 0.75 mgGAE/100 g fw for “Laskava”. This is in correspondence with the fact that the peach peels contain two to three times the concentration of total phenolic compounds compared to the flesh [59,62]. The peach skin is usually neglected by consumers, and repeatedly discarded, even though it is rich in health-promoting phytonutrients. In our study, all “July Lady” extracts exhibited the highest TPC compared to the other varieties. Meanwhile, “Laskava” was the poorest variety in terms of phenolic compounds.

Flavonoids are an important group of phenolic compounds that possess antioxidant potential in addition to their anticarcinogenic, antimicrobial, and cardioprotective properties [66]. In the current investigation, the total flavonoid content varied, among samples, as follows: for WEF from 2.98 ± 0.10 to 15.65 ± 0.06 mgQE/100 g fw; for MEP from 4.70 ± 0.16 to 31.49 ± 0.20 mgQE/100 g fw; and for MEF from 7.86 ± 0.15 to 33.07 ± 0.17 mgQE/100 g fw. Although previous research reported that flavonols can be found mainly in the peach peels [62], the present findings show that a varied distribution between the extracts (peel, flesh, whole fruit) of the different varieties was observed. The lowest in total flavonoids were “Ufo 4” and “Laskava” varieties.

Anthocyanins are a subgroup of flavonoids that are mainly responsible for the coloration in fruits [67]. Cyanidin-3-O-glucoside and cyanidin-3-O-rutinoside are the most common anthocyanins in peaches, while cyanidin3-O-glucoside was only reported in nectarines [41]. The peel is naturally richer in these phytochemicals, especially the red-colored cultivars [68]. In this study, the lowest values were established for the white flesh variety—"Ufo 4”. In the methanolic peel extract of “July Lady”, 7133.6 ± 388.8 µg C3GE/100 g fw was measured, while in “Ufo 4”—547.1 ± 28.1 µg C3GE/100 g fw. The water peach extracts (WEF) of all studied varieties were poorer in anthocyanins compared to the methanolic extracts (MEF). The WEF of “Laskava” contained 62.7 ± 3.8 µg C3GE/100 g fw, while the same extract of “Filina”—849.3 ± 15.3 µg C3GE/100 g fw. The richest MEF among the varieties was the “Filina” one—849.3 ± 15.3 µg C3GE/100 g fw. Similar results were reported by Zhang et al. [69] for 33 peach cultivars (138.16 ± 5.05 µg C3GE/g fw in the methanolic pulp extract). These results are consistent with previous studies reporting the phytochemical content in the peel and pulp of peach fruit [59,68,70,71].

Among the studied phenolic acids, hydroxycinammic acids were the most abundant. As previously reported, chlorogenic acid dominated in all samples and extracts [72]. Besides acting as an antioxidant, chlorogenic acid, together with caffeic acid, are the two major phenolic acids in the epidermis and subtending cell layers of the peach. Their concentrations are especially high in peach genotypes with a high level of brown rot fungus (*Monilinia fructicola*) resistance [72]. Early-ripening varieties (“Filina”, “Ufo 4”, and “Gergana”) seemed to have higher phenolic acid contents. The “Laskava” variety had significantly lower total phenolic acids—WEF-58.0 µg/g fw, MEP-88.5 µg/g fw, and MEF-29.0 µg/g fw.

In fact, fruit phenolics have a role in fruit visual appearance (color), taste (astringency), and health-beneficial antioxidant properties [62]. Tomás-Barberán et al. [62] reported no clear differences between the phenolic content of nectarines and peaches and the color of the flesh of the cultivars. Furthermore, no clear trend in the phenolic content and ripening of the different cultivars was observed [20,30]. This is not the case of the present research where the mid-ripening varieties and the white flesh flat peach had less total phenolic acids, which is in agreement with the results documented by Scordino et al. [73].

The presence of phytochemicals in the studied peaches suggests the existence of antioxidant activity. In addition, various studies have shown good correlation between antioxidant capacity and total phenolic compound content [74,75]. Therefore, four generally accepted methods were used to study it. The three extracts of each sample were analyzed by two free radical scavenging methods (DPPH^•^ and ABTS^•+^), ferric-reducing power (FRAP) and cupric ion-reducing antioxidant capacity (CUPRAC) assays (Table 7.). Different antioxidant capacity analyses for the same samples showed a range of variation. However, by all the methods, the lowest potential was measured in WEF regardless of the variety. The highest antioxidant potential was recorded for the methanol peel extracts of “Filina”, “July Lady”, and “Gergana”, and of the lowest in “Ufo 4” and “Laskava” varieties. Summarizing the data, it emerges that 80% methanol seemed to be more effective in extracting compounds with antioxidant activity.

Following the DPPH methods, significant variations between the extracts and varieties were measured—31.89 ± 0.31 to 728.98 ± 3.74 µMTE/100 g fw. “Filina” and “July Lady” showed the highest DPPH inhibitory potential regarding the methanol extracts, while “Filina” and “Gergana” in regard to the water extract (180.01 ± 1.39 and 53.37 ± 0.30 µMTE/100 g fw, resp.).

ABTS is another method that describes the capacity of neutralizing free radicals. The measured Trolox equivalents of the peel and fruit methanol extracts varied from 126.05 ± 1.70 to 1332.61 ± 6.29 µMTE/100 g fw, and from 112.10 ± 1.30 to 386.17 ± 5.03 µMTE/100 g fw in the water extracts. Both the peel and the fruit methanol extracts of “July Lady”, “Filina”, and “Laskava” showed the highest ABTS inhibitory potential. With respect to the water fruit extracts, the highest values were established again in “July Lady” and “Filina” varieties (352.30 ± 3.73 and 386.17 ± 5.03 µMTE/100 g fw, resp.). The lowest ABTS values in both peel and fruit samples were observed for the “Ufo 4” variety.

Similarly, according to the FRAP and CUPRAC assays, the highest values were established in “July Lady” methanol peel extract (1704.603.7 and 1318.10 ± 11.80 µMTE/100 g fw, resp.), followed by “Filina” and “Gergana” ones. The lowest antioxidant potential was detected in “Laskava” and “Ufo 4” water fruit extracts for both assays—under 130 µMTE/100 g fw. Briefly, a wide variation in the antioxidant potential in the studied varieties was found. Other authors observed differences related to cultivar, tissue, and ripening stage [35]. Lombardo et al. [76] established a high concentration of bioactive compounds with significant antioxidant activity in the early stage of peach development due to their role of plant-protecting agents.

**Figure 2:**
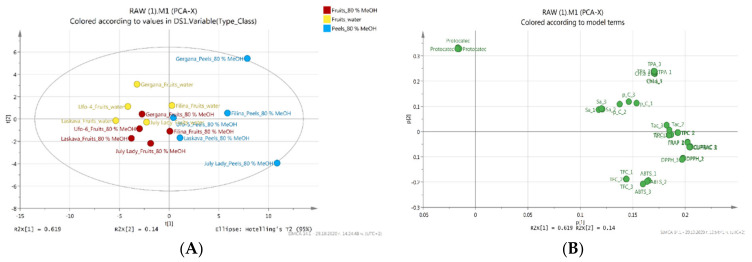
Scores and loading plots of principal components 1 and 2 of the principal component analysis (PCA) results obtained from phytochemical compounds and antioxidant activities in different extracts of peach varieties—(**A**)—score plat and (**B**)—loading plot.

PCA has been the most frequently used clustering technique to determine how one sample is distinct from another. In the present study, principal component analysis of the obtained peach extracts was performed on 12 variables, including TFC, TPC, DPPH, ABTS, FRAP, CUPRAC, total monomeric anthocyanins, individual phenolic acids, and total phenolic acid content (Figure 2). The results showed that the two principal components explained 75.9% of the total variance. The first principal component (PC1) explained 61.9% of the total variance, while PC2 explained 14%. PC1 interpreted the total phenolic acids, total flavonoid content, chlorogenic acid, and ABTS antioxidant activity. PC1 is generally better correlated with the variables than PC2. This is to be expected because PCs are extracted successively, each one accounting for as much of the remaining variance as possible. PC2 was mainly attributed to protocatechuic acid. Not surprisingly, total phenolic content, total monomeric anthocyanin content, and DPPH and FRAP antioxidant activity data are clustered together on the right hand side of the loading plot. These parameters are significantly correlated as evidenced by their Pearson correlation coefficients (data not shown). Total flavonoid content and ABTS antioxidant activity are colocated in a region of the PC space. When the data of both solvents (water and 80% methanol) were plotted, differences were noted. The location of peel extracts of the varieties “Gergana” and “July Lady” on the graph shows a clear distinction, which is evident in the extracts of peels and fruits of the same varieties (Figure 2A).

## 4. Conclusions

The present study could be assumed as the first to establish a comprehensive chemical and nutritional profile of peaches and nectarines (peel, flesh, and both) of different ripening stages. The heterogeneity of the study in terms of peach varieties (ripening stage and type) can better explain the differentiation of results, i.e., carbohydrates (sugars and dietary fibers), amino acid content, and lipids, as well as mineral content, fat-soluble vitamins, carotenoids and chlorophyll, polyphenolic compounds, and the corresponding antioxidant activity. Experiments confirmed that different parts of the peach (peel, flesh) have their own distinct properties. Due to the capacity of the extracts’ solvents, the methanolic extract of the peel seemed to be richer in the studied biologically active substances compared to the fleshy part of the fruit. Peaches, the object of the current study, are a good source of several minerals and vitamins, as well as bioactive compounds of high antioxidant activity, particularly polyphenols and tocopherols. “Filina” was the variety with the richest mineral and carbohydrate content; “Gergana” had the most fiber and minerals; the most carotenoids were contained in “July Lady”, while “Laskava” possessed the most lipids. The current research results indicate many health-promoting properties of peach fruit, pinpointing the unique characteristics of each variety. Researchers can use this study to encourage the daily consumption of peaches.

## Figures and Tables

**Table 1:** Mineral content of different varieties of peach (*Prunus persica* L.) varieties, mg/kg fw *. (* Nitrogen content is expressed as g/kg fw).

Minerals	“Filina”	“Gergana”	“Ufo 4”	“July Lady”	“Laskava”
**Cu**	1.58	0.74	0.48	0.57	1.23
**Fe**	0.64	0.54	0.31	0.26	0.9
**Mn**	0.15	0.17	0.17	0.17	0.17
**Cr**	0.32	0.17	0.5	0.71	0.19
**Zn**	1.35	1.08	0.67	0.75	0.93
**Ca**	0.44	0.63	0.31	<0.15	<0.15
**Mg**	11.26	5.3	3.6	0.93	14.73
**K**	1036.78	974.5	574.67	1179	701.29
**Na**	57.83	36.37	32.16	4.16	20.01
**P**	160.72	101.43	76.77	nd	160
**Pb**	<0.10	<0.10	<0.10	<0.10	<0.10
**Total**	1271.17	1121.03	689.74	1186.8	899.7
N *	1.7	9.6	8.1	6.1	1.3

nd—not detected.

**Table 2:** The amino acid composition of peach (*Prunus persica* L.) varieties (mg/100 g fw).

Variety	“Filina”	“Gergana”	“Ufo 4”	“July Lady”	“Laskava”
**Essential amino acids**					
**Val**	19.94 ± 0.28 ^c^	44.29 ± 0.33 ^b^	75.28 ± 0.55 ^a^	1.58 ± 0.02 ^e^	10.98 ± 1.11 ^d^
**Met**	5.16 ± 0.16 ^d^	54.02 ± 0.38 ^a^	17.34 ± 0.08 ^c^	0.49 ± 0.02 ^e^	24.65 ± 1.23 ^b^
**Lys**	27.01 ± 0.15 ^d^	125.79 ± 1.21 ^b^	157.29 ± 1.12 ^a^	2.25 ± 0.08 ^e^	35.67 ± 0.35 ^c^
**Ile**	17.39 ± 0.25 ^c^	38.82 ± 0.12 ^b^	63.82 ± 0.38 ^a^	1.32 ± 0.07 ^d^	17.14 ± 0.07 ^c^
**Leu**	3.64 ± 0.11 ^c^	7.58 ± 0.09 ^b^	12.04 ± 0.12 ^a^	0.21 ± 0.08 ^e^	0.99 ± 0.01 ^d^
**Phe**	31.53 ± 0.19 ^c^	41.48 ± 0.44 ^b^	68.54 ± 0.65 ^a^	4.88 ± 0.08 ^e^	15.60 ± 0.10 ^d^
**Thr**	29.52 ± 0.19 ^d^	136.77 ± 1.08 ^a^	113.99 ± 1.18 ^b^	1.64 ± 0.05 ^e^	88.07 ± 1.08 ^c^
**Arg**	25.13 ± 0.32 ^c^	55.12 ± 0.13 ^b^	104.26 ± 1.21 ^a^	2.29 ± 0.10 ^e^	20.82 ± 0.81 ^d^
**His**	56.37 ± 0.45 ^c^	62.41 ± 0.11 ^b^	121.37 ± 0.98 ^a^	0.51 ± 0.11 ^e^	29.81 ± 0.82 ^d^
**Non-essential amino acids**					
**Asp**	167.47 ± 1.12 ^a^	nd	nd	nd	9.98 ± 0.38 ^b^
**Ser**	86.67 ± 1.09 ^c^	90.69 ± 1.19 ^b^	181.98 ± 1.46 ^a^	4.10 ± 0.06 ^e^	64.33 ± 1.42 ^d^
**Glu**	41.06 ± 0.92 ^c^	76.59 ± 1.22 ^b^	126.27 ± 1.58 ^a^	7.55 ± 0.04 ^e^	25.63 ± 0.69 ^d^
**Gly**	32.80 ± 1.1 ^a^	11.52 ± 0.26 ^c^	25.97 ± 0.99 ^b^	nd	8.64 ± 0.06 ^d^
**Ala**	100.75 ± 1.19 ^a^	16.00 ± 0.98 ^d^	30.67 ± 1.04 ^b^	1.55 ± 0.18 ^e^	24.41 ± 0.67 ^c^
**Pro**	23.59 ± 0.29 ^c^	43.64 ± 1.29 ^b^	77.09 ± 1.27 ^a^	1.16 ± 0.08 ^e^	4.98 ± 0.33 ^d^
**Cys**	1.10 ± 0.12 ^ab^	2.05 ± 0.78 ^ab^	3.48 ± 0.98 ^a^	0.30 ± 0.13 ^b^	1.25 ± 0.11 ^ab^
**Tyr**	22.82 ± 1.18 ^c^	64.26 ± 1.32 ^b^	98.19 ± 1.44 ^a^	1.70 ± 0.19 ^e^	19.36 ± 0.33 ^d^
**Total**	691.95	971.04	1277.59	31.51	382.95

The data are presented as the mean (*n* = 3) ± S.D. Different letters within each row indicate significant differences between treatments according to Tukey’s test at *p* < 0.05. nd—not determined.

**Table 3:** Protein quality evaluation of peach (*Prunus persica* L.) varieties by amino acid score method (mg/g).

Variety	“Filina”	“Gergana”	“Ufo 4”	“July Lady”	“Laskava”
**Val**	0.51	1.14	1.93	0.04	0.28
**Met + cys**	0.37	3.3	1.22	0.05	1.52
**Lys**	0.6	2.79	3.5	0.05	0.79
**Ile**	0.58	1.29	2.13	0.04	0.57
**Leu**	0.06	0.13	0.2	0	0.17
**Phe + tyr**	2.7	5.56	11.17	0.17	5.56
**Thr**	1.28	5.95	4.95	0.07	3.83
**His**	3.76	4.16	8.09	0.34	1.99

**Table 4:** Carbohydrates (g/100 g fw), dietary fiber (g/100 g fw), total lipids (g/100 g fw), and energy value (kcal) of peach (*Prunus persica* L.) varieties.

Variety	“Filina”	“Gergana”	“Ufo 4”	“July Lady”	“Laskava”
**Carbohydrates**					
**Sucrose**	1.81 ± 0.05 ^c^	1.11 ± 0.09 ^d^	1.91 ± 0.03 ^c^	2.63 ± 0.08 ^b^	4.71 ± 0.05 ^a^
**Glucose**	0.56 ± 0.02 ^c^	0.47 ± 0.03 ^d^	0.41 ± 0.05 ^d^	0.8 ± 0.01 ^b^	1.51 ± 0.02 ^a^
**Fructose**	0.30 ± 0.04 ^bc^	0.26 ± 0.05 ^cd^	0.17 ± 0.01 ^d^	0.37 ± 0.03 ^b^	0.86 ± 0.03 ^a^
**Sorbitol**	0.02 ± 0.01 ^b^	0.02 ± 0.01 ^b^	0.02 ± 0.01 ^b^	0.05 ± 0.01 ^b^	0.11 ± 0.02 ^a^
**Sucrose/Glucose**	3.23	2.36	4.66	3.29	3.12
**Glucose/Fructose**	1.87	1.81	2.41	2.16	1.76
**Total sugars**	2.68 ± 0.03 ^c^	1.85 ± 0.05 ^e^	2.51 ± 0.03 ^d^	3.85 ± 0.05 ^b^	7.19 ± 0.05 ^a^
**Total carbohydrates**	12.42 ± 0.32 ^a^	8.12 ± 0.15 ^d^	6.54 ± 0.25 ^e^	9.49 ± 0.30 ^c^	10.58 ± 0.55 ^b^
**Dietary fiber**					
**Total dietary fiber, TDF**	2.62 ± 0.09 ^b^	3.18 ± 0.10 ^a^	2.05 ± 0.08 ^c^	2.52 ± 0.08 ^b^	2.49 ± 0.04 ^b^
**Insoluble dietary fiber, IDF**	2.03 ± 0.08 ^b^	2.35 ± 0.07 ^a^	1.49 ± 0.07 ^d^	1.70 ± 0.07 ^c^	1.77 ± 0.06 ^c^
**Soluble dietary fiber, SDF**	0.52 ± 0.04 ^c^	0.72 ± 0.03 ^b^	0.55 ± 0.03 ^c^	0.82 ± 0.04 ^a^	0.69 ± 0.03 ^b^
**Total lipids**	1.01 ± 0.08 ^bc^	0.67 ± 0.07 ^c^	0.92 ± 0.12 ^c^	1.38 ± 0.18 ^a^	2.58 ± 0.27 ^a^
**Energy value**	61.53	42.39	39.56	50.5	67.06

The data are presented as the mean (*n* = 3) ± S.D. Different letters within each row indicate significant differences between treatments according to Tukey’s test at *p* < 0.05.

**Table 5:** Carotenoids (µg/g fw), tocopherols (µg/g fw), and total chlorophyll (mg/g fw) content of peach (*Prunus persica* L.) varieties.

Variety /Compound	“Filina”	“Gergana”	“Ufo 4”	“July Lady”	“Laskava”
**Carotenoids**					
Lutein	2.52 ± 0.02 ^d^	3.64 ± 0.03 ^b^	1.60 ± 0.01 ^e^	3.85 ± 0.02 ^a^	2.70 ± 0.02 ^c^
Lycopene	3.15 ± 0.03 ^d^	6.12 ± 0.0.3 ^b^	0.89 ± 0.01 ^e^	23.85 ± 0.09 ^a^	4.45 ± 0.03 ^c^
β-carotene	0.07 ± 0.01 ^d^	6.50 ± 0.02 ^b^	0.04 ± 0.01 ^d^	14.54 ± 0.04 ^a^	0.85 ± 0.01 ^c^
Total carotenoids	5.74	16.26	2.53	42.24	8
**Tocopherols**					
δ-tocopherol	nd	nd	nd	nd	nd
γ-tocopherol	nd	nd	nd	nd	nd
α-tocopherol	2.55 ± 0.02 ^d^	5.06 ± 0.02 ^c^	2.32 ± 0.02 ^d^	8.69 ± 0.02 ^b^	60.40 ± 0.66 ^a^
**Total chlorophyll**					
Chlorophyll (A)	nd	nd	11.79 ± 0.05	nd	nd
Chlorophyll (B)	nd	nd	64.49 ± 0.16	nd	nd
Total chlorophyll (A + B)	nd	nd	76.28 ± 0.22	nd	nd

The data are presented as the mean (*n* = 3) ± S.D. Different letters within each row indicate significant differences between treatments according to Tukey’s test at *p* < 0.05. nd—not determined.

**Table 6:** Total phenolic content (mg GAE/100 g fw), total flavonoids (mgQE/100 g fw), total monomeric anthocyanins (µg cyanidin-3-glucoside (C3GE)/100 g fw), and phenolic acid content (µg/g fw) of peach (*Prunus persica* L.) varieties.

Variety/ Compound	Type of Extract ***	“Filina”	“Gergana”	“Ufo 4”	“July Lady”	“Laskava”
Total polyphenolic content (TPC)	WEF	104.86 ± 1.45 *^c^	37.74 ± 1.24 ^hi^	40.55 ± 0.69 ^hi^	68.38 ± 0.22 ^f^	34.11 ± 0.54 ^i^
	MEP	154.12 ± 0.163 ^a^	133.67 ± 0.59 ^b^	91.61 ± 1.84 ^d^	157.97 ± 0.67 ^a^	78.19 ± 0.75
	MEF	88.11 ± 0.77 ^de^	43.01 ± 0.95 ^h^	34.53 ± 0.43 ^i^	58.65 ± 0.68 ^g^	58.00 ± 0.72 ^g^
Total flavonoid content	WEF	15.65 ± 0.06 ^e^	10.97 ± 0.05 ^g^	2.98 ± 0.10 ^k^	7.18 ± 0.07 ^h^	5.14 ± 0.23 ^ij^
(TFC)	MEP	6.10 ± 0.16 ^i^	23.92 ± 0.4 ^d^	4.70 ± 0.16 ^j^	31.49 ± 0.20 ^a^	13.17 ± 0.34 ^f^
	MEF	33.07 ± 0.17 ^b^	26.49 ± 0.19 ^c^	7.95 ± 0.10 ^h^	4.68 ± 0.11 ^j^	7.86 ± 0.15 ^h^
Total monomeric anthocyanins	WEF	365.6 ± 61.7 ^fg^	117.5 ± 14.5 ^fg^	64.1 ± 3.3 ^g^	99.48 ± 5.16 ^a^	62.7 ± 3.8 ^g^
	MEP	1394.9 ± 45.4 ^d^	6624.8 ± 404.9 ^b^	547.1 ± 28.1 ^ef^	7133.6 ± 388.8 ^a^	3589.5 ± 76.8 ^c^
	MEF	849.3 ± 15.3 ^e^	384.4 ± 26.5 ^fg^	211.0 ± 1.0 ^fg^	228.7 ± 12.2 ^fg^	338.0 ± 13.8 ^fg^
Phenolic acids **						
Protocatechuic acid	WEF	11.1 ± 0.15 ^e^	25.37 ± 0.30 ^a^	12.80 ± 0.10 ^d^	5.50 ± 0.10 ^i^	14.13 ± 0.25 ^c^
	MEP	9.90 ± 0.05 ^f^	18.23 ± 0.23 ^b^	3.23 ± 0.11 ^j^	3.47 ± 0.11 ^j^	11.47 ± 0.25 ^e^
	MEF	7.61 ± 0.10 ^h^	8.73 ± 0.15 ^g^	2.23 ± 0.15 ^k^	1.83 ± 0.11 ^k^	8.33 ± 0.15 ^g^
Chlorogenic acid	WEF	278.1 ± 0.57 ^d^	168.83 ± 1.77 ^h^	149.8 ± 4.00 ^j^	190.23 ± 2.80 ^j^	39.20 ± 0.95 ^n^
	MEP	361.87 ± 3.59 ^c^	738.43 ± 0.81 ^a^	261.57 ± 1.72 ^e^	401.00 ± 1.41 ^b^	54.4 ± 0.95 ^m^
	MEF	197.17 ± 1.81 ^f^	158.2 ± 1.15 ^i^	93.03 ± 0.70 ^l^	131.5 ± 1.64 ^k^	13.40 ± 0.62 ^o^
p-Coumaric acid	WEF	21.80 ± 1.47 ^b^	14.30 ± 0.46 ^cd^	14.40 ± 0.56 ^cd^	12.54 ± 0.43 ^ds^	2.37 ± 0.02 ^f^
	MEP	22.53 ± 0.91 ^b^	21.63 ± 1.03 ^b^	26.23 ± 0.55 ^a^	20.96 ± 0.74 ^b^	16.10 ± 0.40 ^c^
	MEF	12.13 ± 0.80 ^e^	12.93 ± 0.45 ^de^	13.53 ± 0.40 ^de^	11.63 ± 0.49 ^e^	3.70 ± 0.10 ^f^
Sinapic acid	WEF	1.99 ± 0.01 ^k^	2.89 ± 0.02 ^h^	0.49 ± 0.03 ^m^	2.25 ± 0.05 ^ij^	2.18 ± 0.08 ^jk^
	MEP	9.40 ± 0.10 ^a^	8.38 ± 0.07 ^b^	1.45 ± 0.05 ^l^	4.47 ± 0.06 ^g^	7.23 ± 0.06 ^c^
	MEF	2.40 ± 0.10 ^i^	5.38 ± 0.08 ^e^	6.10 ± 0.10 ^d^	1.55 ± 0.05 ^l^	3.29 ± 0.03 ^g^
Total phenolic acids	WEF	313.56 ± 1.70 ^d^	211.39 ± 1.59 ^g^	177.49 ± 4.62 ^i^	210.52 ± 3.03 ^g^	57.89 ± 0.87 ^m^
	MEP	403.70 ± 2.82 ^c^	786.68 ± 1.55 ^a^	292.48 ± 2.14 ^e^	429.90 ± 1.65 ^b^	89.2 ± 0.61 ^l^
	MEF	219.32 ± 2.68 ^f^	185.25 ± 1.38 ^h^	114.9 ± 0.35 ^k^	146.52 ± 1.38 ^j^	28.72 ± 0.77 ^n^

The data are presented as the mean (*n* = 3) ± S.D. Different letters within each row (TPC, TFC, TMA) and within each phenolic acid indicate significant differences between treatments according to Tukey’s test at *p* < 0.05. ** Gallic acid, caffeic acid, ferulic acid, rosmarinic acid, cichoric acid, and cinnamic acid were not detected in any extract. *** WEF—water peach extract, MEP—ultrasonic peach extract, MEF—methanolic peach extract

**Table 7:** Antioxidant activity of peach (*Prunus persica* L.) varieties (µMTE/100 g fw).

Variety/ Compound	Type of Extract	“Filina”	“Gergana”	“Ufo 4”	“July Lady”	“Laskava”
DPPH	WEF	180.01 ± 1.39 ^f^	77.66 ± 0.95 ^i^	48.01 ± 0.40 ^k^	53.37 ± 0.30 ^k^	31.89 ± 0.31 ^l^
	MEP	542.76 ± 3.64 ^b^	370.04 ± 5.04 ^c^	132.03 ± 2.56 ^g^	728.98 ± 3.74 ^a^	376.39 ± 1.14 ^c^
	MEF	239.13 ± 1.37 ^d^	73.71 ± 0.39 ^i^	112.78 ± 0.92 ^h^	190.75 ± 0.75 ^e^	64.66 ± 2.9 ^j^
ABTS	WEF	386.17 ± 5.03 ^e^	196.62 ± 2.65 ^f^	112.10 ± 1.30 ^g^	352.30 ± 3.73 ^e^	197.45 ± 4.23 ^f^
	MEP	1125.21 ± 13.01 ^b^	568.18 ± 6.05 ^d^	428.78 ± 4.34 ^e^	1332.61 ± 6.29 ^a^	1153.87 ± 5.14 ^b^
	MEF	532.79 ± 3.46 ^d^	126.05 ± 1.70 ^fg^	201.17 ± 0.67 ^f^	631.73 ± 4.91 ^c^	530.88 ± 7.21 ^d^
FRAP	WEF	423.30 ± 4.70 ^f^	169.40 ± 1.20 ^k^	110.90 ± 0.90 ^m^	340.10 ± 2.9	91.40 ± 1.10 ^m^
	MEP	963.0 ± 4.20 ^c^	1101.10 ± 4.10 ^b^	668.30 ± 6.10 ^d^	1704.603.7 ^a^	220.60 ± 6.3 ^i^
	MEF	590.0 ± 1.50 ^e^	175.20 ± 1.40 ^k^	207.70 ± 1.50 ^j^	390.80 ± 5.10 ^g^	156.20 ± 0.8 ^l^
CUPRAC	WEF	569.40 ± 8.30 ^f^	258.40 ± 0.00 ^i^	131.90 ± 0.00 ^k^	351.50 ± 5.10 ^h^	121.10 ± 1.50 ^k^
	MEP	936.10 ± 26.40 ^b^	942.20 ± 12.70 ^b^	615.80 ± 10.10 ^e^	1318.10 ± 11.80 ^a^	811.70 ± 12.10 ^c^
	MEF	681.90 ± 3.60 ^d^	264.40 ± 2.70 ^i^	256.0 ± 3.50 ^i^	446.0 ± 6.40 ^g^	192.70 ± 2.50 ^j^

The data are presented as the mean (*n* = 3) ± S.D. Different letters within each antioxidant assay indicate significant differences between treatments according to Tukey’s test at *p* < 0.05; Ferric Reducing Antioxidant Power (FRAP), Cupric Reducing Antioxidant Capacity (CU-PRAC), 2,2-Diphenyl-1-picrylhydrazyl (DPPH) and 2,2′-Azinobis-(3-Ethylbenzothiazoline-6-Sulfonic Acid (ABTS); WEF—water peach extract, MEP—ultrasonic peach extract, MEF—methanolic peach extract.

## Data Availability

The data presented in this study are available on request from the corresponding author.
